# [^18^F]FE-PE2I PET is a feasible alternative to [^123^I]FP-CIT SPECT for dopamine transporter imaging in clinically uncertain parkinsonism

**DOI:** 10.1186/s13550-022-00930-x

**Published:** 2022-09-07

**Authors:** Lisbeth Marner, Kirsten Korsholm, Lasse Anderberg, Markus N. Lonsdale, Mads Radmer Jensen, Eva Brødsgaard, Charlotte L. Denholt, Nic Gillings, Ian Law, Lars Friberg

**Affiliations:** 1 Department of Clinical Physiology and Nuclear Medicine, Copenhagen University Hospital Bispebjerg, Bispebjerg Bakke 23, Copenhagen, Denmark; 2 Department of Clinical Medicine, University of Copenhagen, Copenhagen, Denmark; 3 Department of Clinical Physiology, Nuclear Medicine and PET, Copenhagen University Hospital Rigshospitalet, Copenhagen, Denmark

**Keywords:** Brain, Parkinson’s disease, Positron emission tomography, Cerebral, Neurodegenerative, DaTscan, Diagnostic accuracy

## Abstract

**Background:**

Dopamine transporter (DAT) imaging of striatum is clinically used in Parkinson’s disease (PD) and neurodegenerative parkinsonian syndromes (PS) especially in the early disease stages. The aim of the present study was to evaluate the diagnostic performance of the recently developed tracer for DAT imaging [^18^F]FE-PE2I PET/CT to the reference standard [^123^I]FP-CIT SPECT.

**Methods:**

Ninety-eight unselected patients referred for DAT imaging were included prospectively and consecutively and evaluated with [^18^F]FE-PE2I PET/CT and [^123^I]FP-CIT SPECT on two separate days. PET and SPECT scans were categorized independently by two blinded expert readers as either normal, vascular changes, or mixed. Semiquantitative values were obtained for each modality and compared regarding effect size using Glass’ delta.

**Results:**

Fifty-six of the [^123^I]FP-CIT SPECT scans were considered abnormal (52 caused by PS, 4 by infarctions). Using [^18^F]FE-PE2I PET/CT, 95 of the 98 patients were categorized identically to SPECT as PS or non-PS with a sensitivity of 0.94 [0.84–0.99] and a specificity of 1.00 [0.92–1.00]. Inter-reader agreement for [^18^F]FE-PE2I PET with a kappa of 0.97 [0.89–1.00] was comparable to the agreement for [^123^I]FP-CIT SPECT of 0.96 [0.76–1.00]. Semiquantitative values for short 10-min reconstructions of [^18^F]FE-PE2I PET/CT were comparable to longer reconstructions. The effect size for putamen/caudate nucleus ratio was significantly increased using PET compared to SPECT.

**Conclusions:**

The high correspondence of [^18^F]FE-PE2I PET compared to reference standard [^123^I]FP-CIT SPECT establishes [^18^F]FE-PE2I PET as a feasible PET tracer for clinical use with favourable scan logistics.

**Supplementary Information:**

The online version contains supplementary material available at 10.1186/s13550-022-00930-x.

## Introduction

Parkinson’s disease (PD) is the second most frequent neurodegenerative disorder, trailing only Alzheimer’s disease [1]. The primary symptoms can include hypokinesia, tremor, rigidity and postural instability, may start already in the forties, and frequently interfere with the patient’s work status and quality of life for both patient and relatives [2]. In addition, many patients develop sleep disorders, as well as autonomous and cognitive disturbances [3].

The pathophysiology of PD is abnormal deposition of α-synuclein leading to dopaminergic cell loss in the nigrostriatal pathway and consequent loss of dopaminergic axons to the striatum [4]. Functional imaging of the dopamine transporter (DAT), located in the presynaptic neuronal projections, shows decreased signal early in PD and is decreased in neurodegenerative parkinsonian syndromes (PS) [5]. The diagnosis of PD is based on clinical criteria, although brain imaging may aid in settling the diagnosis, as normal dopamine transporter imaging is considered an exclusion criterion for Parkinson’s disease [6]. For decades, DAT has been visualized with either [^123^I]2β-carboxymethoxy-3beta-[4-iodophenyl]tropane ([^123^I]β-CIT) or [^123^I]N-ω-fluoropropyl-2β-carbomethoxy-3β-(4-iodophenyl)nortropan ([^123^I]FP-CIT) using SPECT. The increasing availability of PET scanners with their attractive higher resolution has led to the development of a fluorinated PET tracer, [^18^F](E)-N-(3-iodoprop-2-enyl)-2β-carbofluoroethoxy-3β-(4'-methyl-phenyl) nortropane ([^18^F]-FE-PE2I) for DAT [7]. [^18^F]FE-PE2I has suitable affinity for DAT and low affinity for other monoaminergic transporters—in contrast to [^123^I]FP-CIT, which also binds to the serotonin transporter [7, 8]. Previously, PE2I has been labelled with ^123^I and evaluated using SPECT [9], and a blocking study confirmed that PE2I does not bind significantly to the serotonin transporter [10]. [^18^F]FE-PE2I binding is unaffected by anti-Parkinsonian drugs in rats [11] and a dopamine transporter blocking study with Modafinil supports the use of [^18^F]FE-PE2I as a selective dopamine transporter marker [12]. Labelling with ^11^C shows slow and less favourable kinetics [13–15]. In 2012, [^18^F]FE-PE2I showed promising results in 10 healthy subjects [16] and this was substantiated by Shingai and co-workers [17] who found an age-related decline in DAT density of approximately 7.5% per decade in the caudate and putamen and 3.4% in substantia nigra. Reproducibility over time of PET measurements with [^18^F]FE-PE2I demonstrated good agreement in twelve healthy male subjects[18] and later in nine PD patients [19], making [^18^F]FE-PE2I PET feasible for studying long-term alterations in DAT availability. Based on dynamic [^18^F]FE-PE2I PET scans in PD and healthy subjects, Sonni et al. [20] suggested that static imaging during early peak (17–42 min after injection) could be used as a simplified quantification method compared to the full quantification using long dynamic acquisitions. Use of 20-min static [^18^F]FE-PE2I imaging during early peak was subsequently validated by Brumberg et al. for discriminative power and for longitudinal studies in 33 PD patients and 24 healthy subjects [21].

Several studies have investigated the use of [^18^F]FE-PE2I in substantia nigra and report a less pronounced reduction of [^18^F]FE-PE2I levels compared to striatum [22–24]. Repeatability and reliability have been shown to be very high in striatum with a variability of 5–7% and an intraclass coefficient of 0.91, but only modest in substantia nigra due to the smaller size and DAT density [19]. The effect size for discrimination between PD patients and healthy controls using [^18^F]FE-PE2I PET as compared to [^123^I]FP-CIT SPECT was found to be at least as good by Mo et al. [25] in 22 presumed early-stage PD patients and 28 healthy controls.

[^123^I]FP-CIT SPECT is still the most used radioligand and thus the reference standard for DAT imaging. However, no studies have compared [^18^F]FE-PE2I PET with [^123^I]FP-CIT SPECT, in a large cohort of unselected patients.

The overall aim of this project was to compare the clinical head-to-head performance of [^18^F]FE-PE2I PET/CT and [^123^I]FP-CIT SPECT for DAT imaging in the diagnostic work-up in an unselected cohort of out-patients with clinical features of parkinsonism. We hypothesized that [^18^F]FE-PE2I PET/CT would be non-inferior in the separation of patients with and without pathological DAT availability compared to [^123^I]FP-CIT SPECT). Thus, we aimed to evaluate the use of [^18^F]FE-PE2I PET as a sound clinical tool for imaging large cohorts of patients for DAT availability.

## Methods

We prospectively and consecutively included patients in the diagnostic work-up for Parkinsonism at neurological departments or outpatient neurological clinics referred for DAT imaging at the Department of Clinical Physiology and Nuclear Medicine, Copenhagen University Hospital Bispebjerg from October 2017 to August 2018. Exclusion criteria were severe motor or cognitive disabilities, and more than 2 h of transport time to avoid additional scanning procedures for this vulnerable group. Patients were asked whether they were undergoing treatment with selective serotonin reuptake inhibitors or amphetamine-like medication, but this was not an exclusion criterion. Consents to participate were obtained from all individuals after receiving oral and written information according to national regulations, and the study was registered at the The Committees on Health Research Ethics, Capital Region of Denmark (ID: 17,026,292) that waived need for approval. All data were handled according to regulations by The Danish Data Protection Agency.

### ***Radiosynthesis of [***^***18***^***F]FE-PE2I***

A one-step, one-pot radiosynthesis was used for the production of [^18^F]FE-PE2I based on the method reported by Stepanov and co-workers [26]. Briefly, nucleophilic substitution of a tosyl group with [^18^F]fluoride was followed by high performance liquid chromatography (HPLC) purification (HPLC column:(Onyx™ Monolithic C-18, 100 × 10 mm; flow: 5.5 mL/min). The [^18^F]FE-PE2I containing fraction was collected through a 0.22 µm sterile filter directly in the final product vial containing 15 ml sterile sodium phosphate buffer, thus providing the final product solution (23 mL) containing approximately 6% ethanol. Further details are reported elsewhere [27].

### ***[***^***123***^***I]FP-CIT acquisition, reconstruction and semiquantitative analysis***

**Fig. 1:**
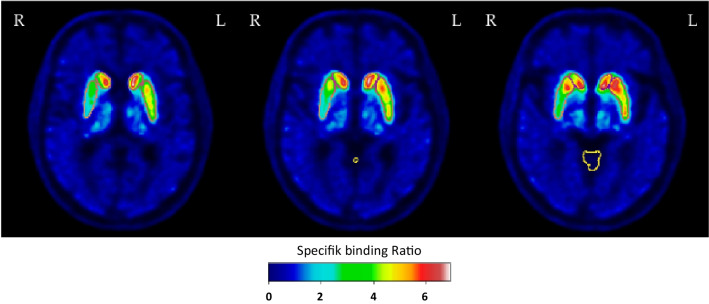
Delineations of anatomical structures. Top row: Five regions of interest (ROI) representing caudate nucleus and putamen bilaterally as well as the occipital lobe were placed on a fused section of the [^123^I]FP-CIT SPECT. Bottom: Three consecutive axial slices (superior—> inferior) of a [^18^F]FE-PE2I PET dataset in MNI space illustrating the automated volume of interest (VOI) delineation of putamen, caudate nucleus and cerebellar grey matter

Patients received 200 mg of sodium perchlorate i.v. 10–15 min before the radiotracer injection in order to block uptake of ^123^I in the thyroid. The SPECT scan was performed 3 h after injection of 185 MBq (5 mCi) [^123^I]FP-CIT and SPECT image acquisition of 30 min was carried out with a PRISM 3000XP (Marconi, Phillips) triple-headed gamma camera equipped with low-energy, ultra-high-resolution fan-beam collimators. SPECT acquisitions were performed using a full 360° rotation. A ^153^Gd source was used for transmission scan. Image reconstruction was performed using iterative reconstruction with corrections for scatter and non-uniform attenuation. The number of iterations was 20 for the transmission data and 4 for the emission data. Pixel size after reconstruction was 3.1 mm in each direction. A 3D low-pass filter (cut-off 0.40, order 4.0) was employed and the data reformatted in oblique slices along the orbitomeatal line with a slice thickness of 6.2 mm. Semiquantitative analysis was performed as previously described [28, 29]. The slice with the highest maximum pixel value was selected and the neighbouring slice with the highest maximum pixel value. The two selected slices were added yielding a single, 12.4 mm thick slice, which was used for further analysis. Four regions of interest (ROI) representing striatum (caudate nucleus and putamen) bilaterally were placed. The shape and size of the ROIs were unchanged, but the ROIs could be rotated and translated to fit the location of the basal ganglia (Fig. 1). Further, a fixed region for non-specific uptake was placed in the occipital lobe as this is a standard DAT SPECT reference region visible within the same slice. The set of these five regions was created to mimic the ROIs defined in the initial clinical verification of the [^123^I]FP-CIT [30]. The ratio of specific striatal and putamen uptake to non-specific uptake, the specific binding ratio (SBR), and the putamen-to-caudate ratio were calculated.

### ***[***^***18***^***F]FE-PE2I acquisition and reconstruction***

PET acquisition was performed on a separate day in a Discovery 710 or MI PET/CT (GE Healthcare, Milwaukee, USA). Patients received a median dose of 192 MBq [^18^F]FE-PE2I (range 40–239 MBq), and 17 min after administration, a 25-min list-mode PET acquisition was performed. All PET scans were preceded by a low-dose CT for attenuation correction and a diagnostic CT-scan. Data were reconstructed into 3D datasets using a commercial implementation (“Q.Clear”) of a block-sequential regularized expectation maximization algorithm with a regularization parameter “β” of 250. In addition to the recommended interval (17–42 min [20]), a second dataset was reconstructed covering the interval from 30–40 min. The two datasets were compared to evaluate if a shorter scan time is feasible without loss of diagnostic accuracy. Table 1 lists practical differences between the clinical setups/workflows on SPECT and PET.

**Table 1:** Comparison between the two clinical setups/workflows

	[^123^I]FP-CIT SPECT	[^18^F]FE-PE2I PET (30–40-min protocol)
Half-life of tracer (transport logistics)	13 h 13 min	1 h 50 min
Patient time	4 h 15 min	1 h 30 min
Time in scanner	30 min	15 min
Staff time	1 h 50 min	1 h 15 min
Scanner	Short moving bore	Long narrow bore
Pretreatment to block thyroid ^123^I uptake	Yes	No
Radiation exposure*	185 MBq / 4.6 mSv	185 MBq /4.3 mSv
Sensitivity to SSRI treatment	Yes [38]	Insignificant [11]
Approximate spatial resolution	10 mm	5 mm

*SSRI* selective serotonin reuptake inhibitors

Time estimates are based on logistics in our high throughput clinic with 20–30 patients/week

*Based on an effective dose of 0.025 mSv/MBq for [^123^I]FP-CIT[38] and 0.023 mSv/MBq for [^18^F]FE-PE2I[44]

### ***[***^***18***^***F]FE-PE2I volumes of interest***

Delineation of volumes of interest (VOI) on static [^18^F]FE-PE2I PET images was performed using an in-house developed automated segmentation algorithm targeting the caudate nuclei, putamina and cerebellar grey matter including vermis in order to compute imaging metrics comparable to [^123^I]FP-CIT SPECT. Cerebellum has previously been validated as a reference region without specific binding in an autoradiographic blocking study using [^125^I]PE2I [31] and was therefore chosen instead of the occipital lobe used for SPECT. These metrics were the SBR’s of the caudate and putamen relative to cerebellar grey matter and the putamen/caudate ratios for each hemisphere. The automatic segmentation method was based on an in-house created atlas with delineations of the above structures from 37 healthy elderly subjects using Joint Label Fusion [32]. These subjects all had matching CT and high resolution T1-weighted MRI which allowed for the target structures to be segmented from MRI using FreeSurfer [33] and transferred rigidly onto the co-registered CT scans. The cerebellar grey matter VOIs were used directly, while the putamen and caudate delineations were filtered with a 6 mm Gaussian kernel to mimic PET resolution. The resulting segmentations were manually corrected when necessary and verified by a nuclear medicine expert specialized in neuroimaging (IL). Individual delineations from the 37 subjects were transformed to MNI space constituting the templates for the automatic segmentation.

For each study patient, the brain was initially extracted from CT using Brain Extraction Tool [34] of the FSL software suite [35] after thresholding and smoothing [36] and was affinely registered to an MNI template using correlation ratio as cost function. Applying the STEPS algorithm [37] (Markov Random field prior value: 5, kernel size: 5), the initial segmentations of putamen, the caudate nucleus, and cerebellar grey matter were applied. To obtain the final segmentations, the PET image was rigidly registered to the CT image, and the transformation aligning CT image to CT template was applied to the resulting PET image. The resulting spatially normalized PET image was then given as input to the Joint Label Fusion algorithm as implemented in ANTs [32] with a search and patch radius of 5 (Fig. 1). All quantitative measurements were completed in native space after reverse transformation. Median values were used to reduce the influence of voxels with extreme values.

### Interpretation of images

SPECT scans were categorized as either normal, PS, vascular, or mixed independently by two experienced specialists in nuclear medicine with 10 and 25 years of experience in neuroimaging, respectively (KK,LF). As PET is the new modality, the scans were categorized into the same classifications by two additional readers with 5 and 15 years of experience in neuroimaging (MRJ, LM) to assess interreader agreement. Reading of images was based on visual assessment in accordance to practice guidelines [38, 39], blinded for the results of the other reader and the other ligand. For SPECT assessment, semiquantitative measures were incorporated in the clinical reading as it has previously been described to influence clinical interpretation [40] and structural changes on CT/MRI preceding the SPECT scan were taken into account if available mimicking clinical routine. Specific attention was paid to structural lesions in the basal ganglia and to the occipital lobe that served as reference region. Clinical cut-off values for normal and abnormal binding were not available for [^18^F]FE-PE2I and thus semiquantitative values were not included in the interpretation. Although six of the patients received injected doses below 100 MBq, the readers deemed all the scans to be of diagnostic quality for visual interpretation. Structural changes on the CT performed with the PET scan were included in the visual assessment of [^18^F]FE-PE2I as described above. Neurodegenerative disease was suspected in case of significant posterior-anterior gradient and left–right asymmetry, while cerebrovascular cause was suspected in case of larger infarctions on structural imaging co-localized with reduction in tracer uptake. In case of disagreement between readers and modalities, consensus reading was performed.

### Statistics

A significance level of 0.05 was used throughout. Cohen’s kappa was used to compare agreement between modalities, and Fleiss’ kappa was used to compare agreement between readers using SPSS (IBM SPSS Statistic version 25). For analyses of semiquantitative values, the six patients with injected activity below 100 MBq were excluded. The effect sizes for discrimination between abnormal and normal scans using putamen/caudate nucleus ratio and putamen SBR for the worst hemisphere were calculated for the two PET reconstructions (30–40 min versus 17–42 min) and for SPECT versus PET using Glass’ ∆ calculated as (Mean_abnormal_-Mean_normal_)/SD_normal_ (R Core Team, 2017; R Foundation for Statistical Computing, Vienna, Austria; https://www.R-project.org). The study complies with the standards of reporting diagnostic accuracy studies (Additional file 1: Table S1).

## Results

**Fig. 2:**
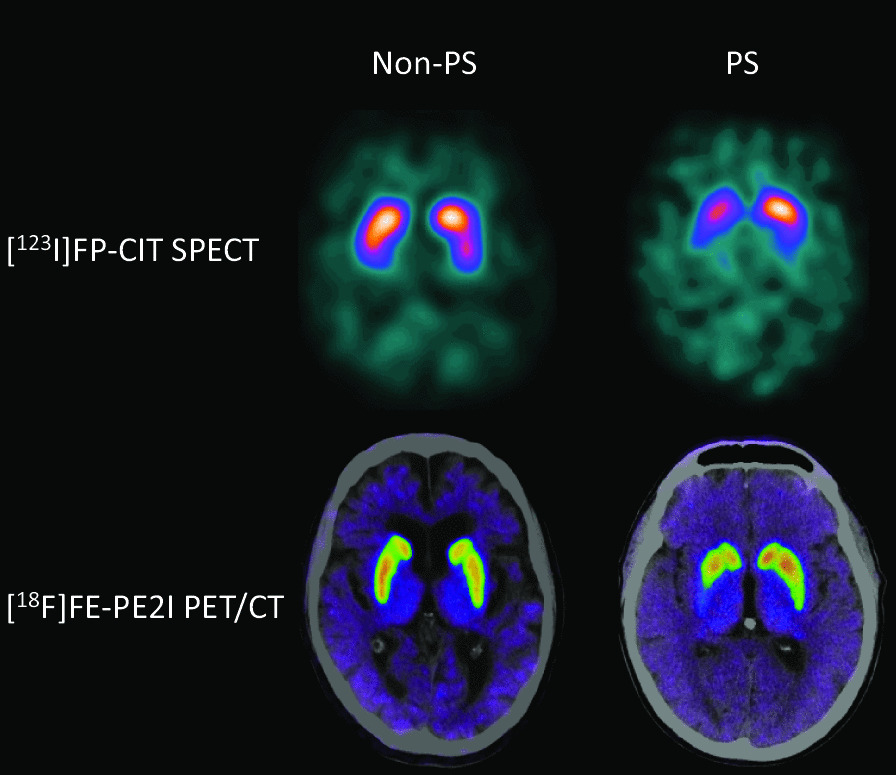
Sample images of two patients evaluated with [^123^I]FP-CIT SPECT and [^18^F]FE-PE2I PET. PS: Parkinsonian syndromes

Ninety-nine unselected patients were recruited (31/68 females/males, median age 72 years, range 36–87 years). One female was excluded due to the inability to perform both scans. Six patients received [^18^F]FE-PE2I with an activity dose below 100 MBq (1 patient below 70 MBq). The low doses were due to tracer production issues and to tracer sticking to the tubes. All PET scans were judged as being of sufficient diagnostic quality by all readers (for image examples, see Fig. 2). The median time-interval between the scanning procedures was 16 days (range 1–64 days). None of the patients were deemed to have potential therapeutic interactions with the tracers. Forty-two of the [^123^I]FP-CIT SPECT scans were rated normal, and 56 abnormal, of these 52 were due solely to neurodegeneration and 4 were due to vascular changes, see Table 2. Using [^123^I]FP-CIT SPECT as the reference standard, [^18^F]FE-PE2I PET revealed an overall sensitivity [95% confidence interval (C.I.)] of 0.94 [0.84–0.99] and a specificity of 1.00 [0.92–1.00] for PS vs non-PS. Using kappa statistics, the agreement of PS vs non-PS, Cohen’s kappa was 0.94 [0.87–1.00] and agreement for subgroup diagnoses (Table 2) between modalities revealed a kappa of 0.85 [0.76–0.94]. Inter-reader agreement for 4 readers for reading of [^18^F]FE-PE2I PET revealed a Fleiss’ kappa of 0.88 [0.82–0.94] and 0.97 [0.89–1.00] for subgroup comparison and for PS vs non-PS, respectively, which was comparable to the agreement for two readers for [^123^I]FP-CIT SPECT with a Fleiss’ kappa of 0.90 [0.72 1.00] and 0.96 [0.76–1.00], respectively. Of the eight patients with disagreement between modalities, four could be attributed to vascular changes visible on the CT obtained during PET imaging, as no prior structural imaging was available for the [^123^I]FP-CIT SPECT reading (Additional file 2: Table S2). Three of the remaining disagreements were between normal and cerebrovascular changes, i.e. none of the modalities reported neurodegenerative disease. In only one patient (#6 in Additional file 2: Table S2), disagreement was not related to structural or vascular changes. Follow-up three years later was available for this patient and revealed an unsettled diagnosis with continuous fall-associated gait disturbances, increasing cognitive symptoms, a repeated normal [^18^F]FE-PE2I PET and an [^18^F]FDG PET not suggesting neurodegenerative disease. Effect of Levodopa was at first admission described to be uncertain. For reading of the [^123^I]FP-CIT SPECT scans, a CT or MRI was available in 49 patients (50%), and in 7 of these an abnormality was noted in close proximity (2 cm) to the basal ganglia (e.g. sequelae after lacunar infarction). In four of these patients there was an impact on the final diagnosis due to the vascular changes (Table 2). In the 49 patients without prior structural imaging, CT obtained during [^18^F]FE-PE2I PET/CT revealed an abnormality in 13 CT scans of which only the four previously mentioned scans with disagreement influenced the visual assessment (Additional file 2: Table S2).

**Table 2:**
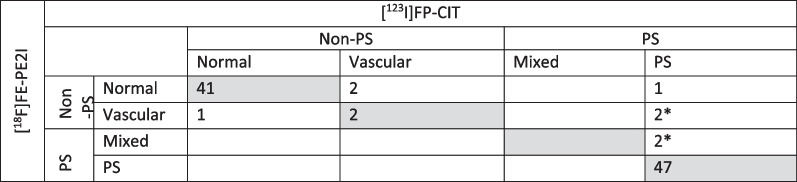
Agreement between [^123^I]FP-CIT and [18F]FE-PE2I

*PS* Parkinsonian syndromes

Measurement of agreement kappa: 0.85 [95% C.I.: 0.76–0.94]. Agreement of normal vs abnormal, kappa: 0.92 [95% C.I.: 0.84–1.00]

*No prior structural imaging available and interpretation of vascular changes based on CT performed in combination with PET

**Fig. 3:**
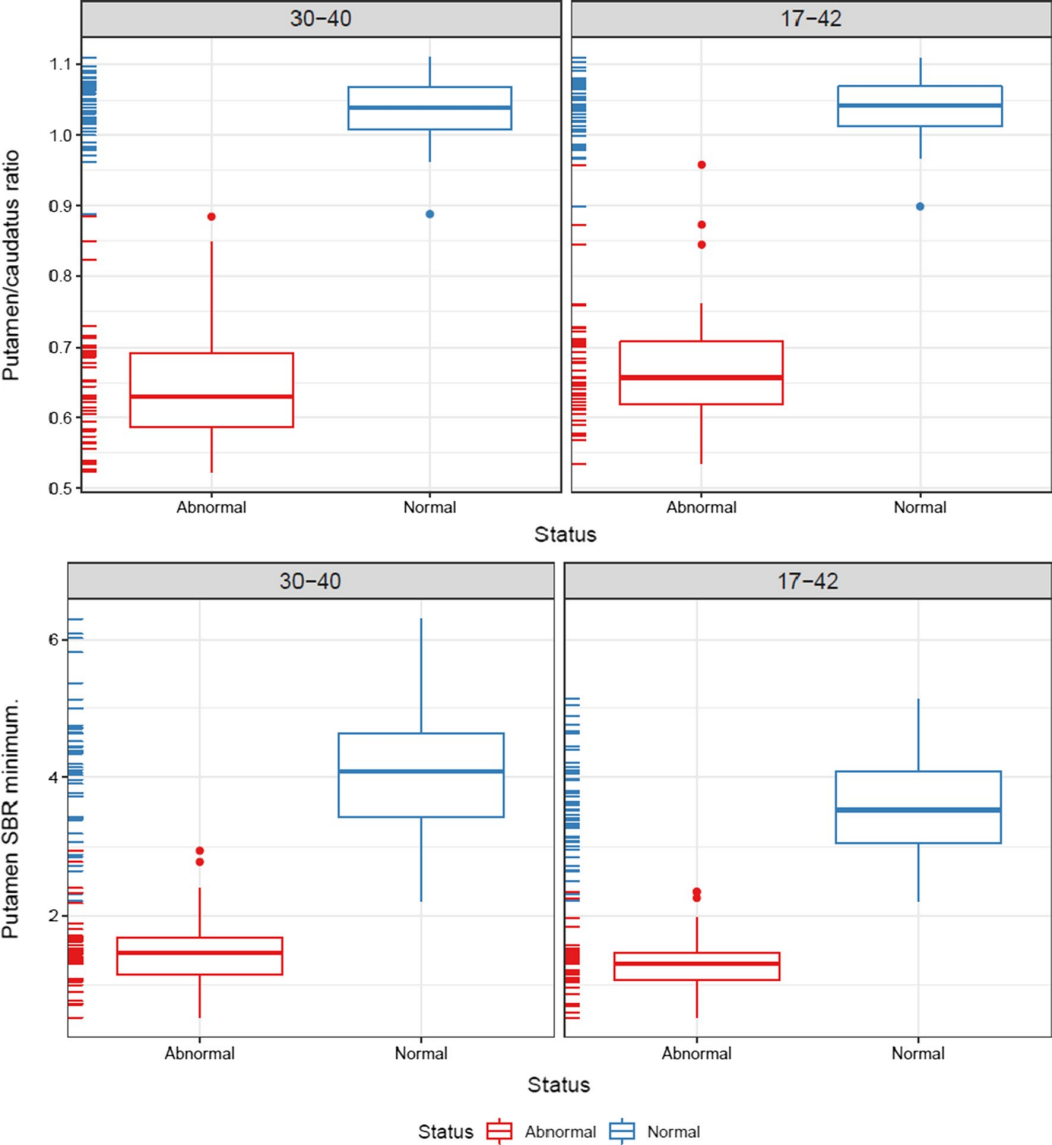
Short versus longer acquisition protocol. Box-and-whisker plots of putamen/caudate nucleus ratio (top) and SBR for putamen (bottom; lowest SBR of right/left hemisphere) for 92 patients illustrating the difference between normal and abnormal patients. Six patients were excluded due to injected dose below 100 MBq. Left column: 30–40-min reconstruction, right column: 17–42 min. No significant difference in the ability to separate abnormal and normal scans was found

**Fig. 4:**
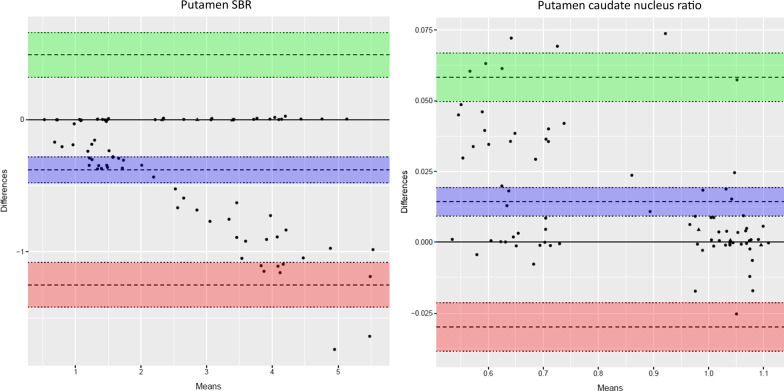
Short versus longer acquisition protocol. Bland–Altman plots of 17–42-min reconstructions versus 30–40-min reconstructions for same patients as in Fig. 3. Triangles represent patients with discrepancy between PET and SPECT reading. The purple area represent the mean difference with 95% confidence limits (C.I.), the green area represent the upper limit of agreement with 95% C.I., and the red area the lower limit of agreement. Please note the relative higher values for putamen SBR (left) using the shorter reconstruction with 95% of patients being within 25% difference. Putamen/caudate nucleus ratio (right) show similarly relatively higher values but the difference is smaller and within 10%. The difference stress the need for using the same acquisition protocol in a future normal database for comparison

**Fig. 5:**
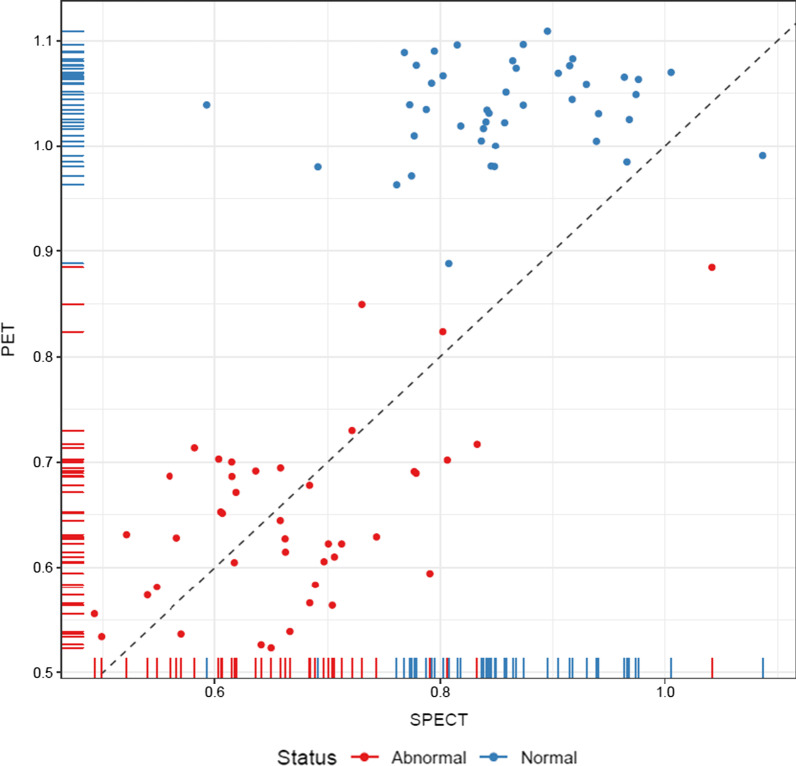
PET versus SPECT. The lowest putamen/caudate nucleus ratio of the two hemispheres for each subject, PET plotted against SPECT. The dotted line is the line of identity. Red circles are patients categorized as abnormal while blue are normal. Note that delineation and semiquantitative measure are obtained with different methods for PET and SPECT

**Fig. 6:**
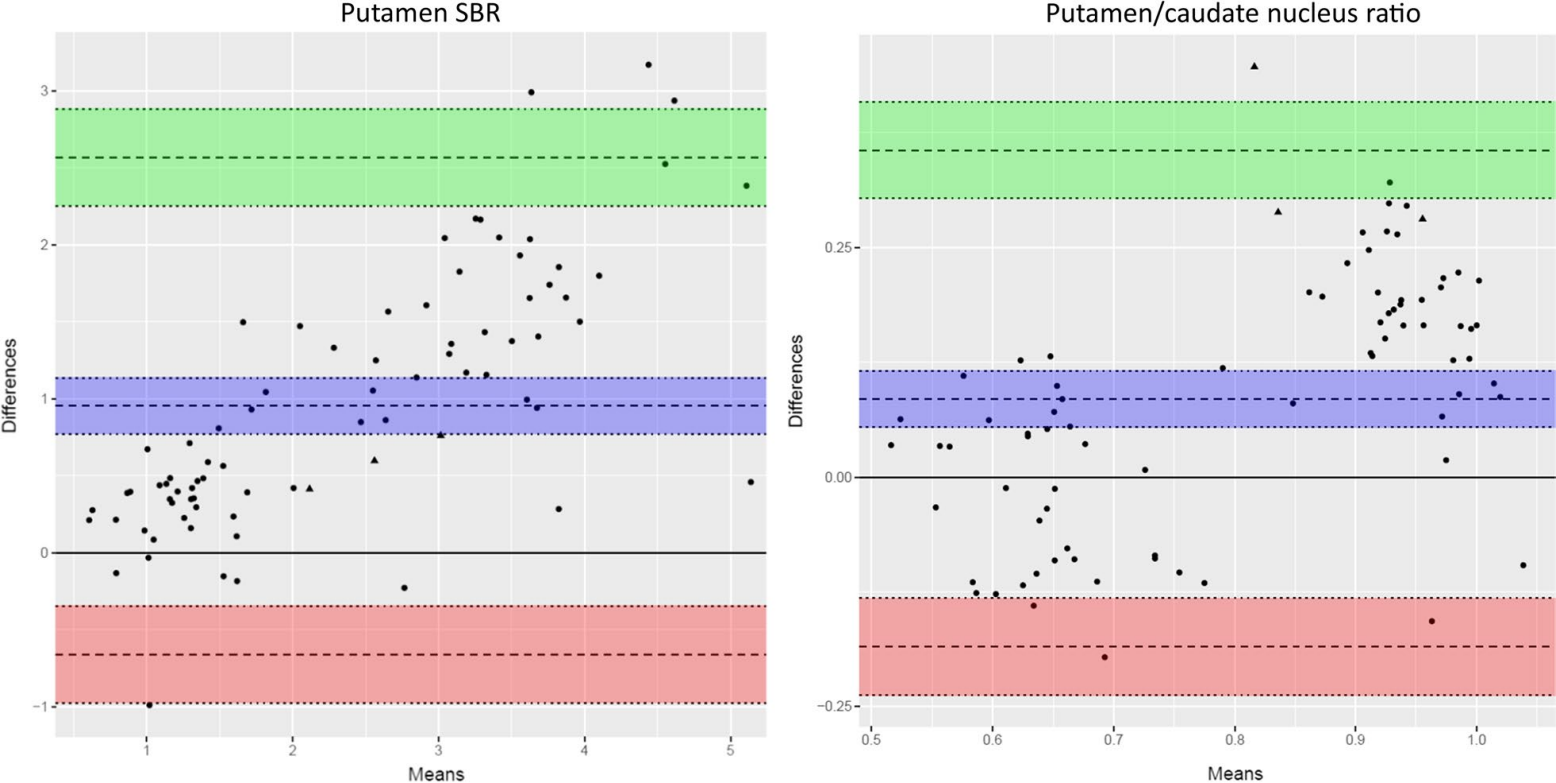
PET versus SPECT Bland–Altman plots. Same data as in Fig. 5 shown as Bland–Altman plots. Triangles represent patients with discrepancy between PET and SPECT reading. The purple area represent the mean difference with 95% confidence limits (C.I.), the green area represent the upper limit of agreement with 95% C.I., and the red area the lower limit of agreement. As expected PET show relative higher values for putamen SBR (left) for PET compared to SPECT with 95% of patients being within 50% difference. Putamen/caudate nucleus ratio (right) show similarly relatively higher values but the difference is smaller and within 35%

The automated VOI delineation of the [^18^F]FE-PE2I PET scans performed well (Fig. 1). Visually, the 30–40-min reconstructed images were qualitatively acceptable as compared to the 17–42-min reconstructions. The semiquantitative analysis with putamen/caudate nucleus ratio and putamen SBR separated healthy and affected persons comparably (Figs. 3 and 4). The effect sizes calculated as Glass’ ∆ were − 8.64[− 10.63; − 6.65] and − 8.44[− 10.39; − 6.47] for 30–40 min and 17–42 min, respectively, for putamen/caudate nucleus ratio and − 2.71[− 3.40; − 2.02] and − 3.01[− 3.76; − 2.26] for putamen SBR. In the following, the 30–40-min reconstruction is used. Figures 5 and 6 shows the relationship between the putamen/caudate nucleus ratio for SPECT and PET. The effects sizes for separating normal from abnormal scans were higher for PET with − 8.64[− 10.63; − 6.65] compared to SPECT with − 2.22[− 2.91; − 1.52] (*p* = 0.0005) for putamen/caudate nucleus ratio and comparable for putamen SBR with − 2.71[− 3.40; − 2.02] and − 2.52[− 3.17; − 1.87] (*p* = 0.82) for PET and SPECT, respectively (Additional file 3: Fig. S1).

## Discussion

We present a head-to-head comparison of [^18^F]FE-PE2I PET to the present reference standard [^123^I]FP-CIT SPECT in a large unselected cohort of 98 patients referred for DAT imaging for uncertain Parkinson’s disease or neurodegenerative parkinsonian syndrome. The study has the largest cohort of patients to date and the findings justify the use of [^18^F]FE-PE2I PET as equivalent to [^123^I]FP-CIT SPECT, as we found very high sensitivity and specificity of 0.94 [0.84–0.99] and 1.00 [0.92–1.00] for PS vs. non-PS using [^123^I]FP-CIT SPECT as reference standard and high agreement between modalities with a kappa of 0.85 [C.I.: 0.76–0.94] for categorizing into four diagnoses (“normal”, “PD”, “cerebrovascular changes”, and “mixed”) (Table 2). The inter-reader agreement for PET was also non-inferior with a Fleiss’ kappa of 0.97 [0.89–1.00] as compared to 0.96 [0.76–1.00] for SPECT for PS vs non-PS. Of the eight patients that differed in categorization, four could be attributed to vascular changes seen on the CT obtained during PET imaging. These findings are in line with earlier studies comparing [^18^F]FE-PE2I PET to [^123^I]FP-CIT SPECT in 22 presumed PD patients compared to 28 healthy subjects [25]. Further, we found an increased effect size of the semiquantitative putamen/caudate nucleus ratio, but not for putamen SBR for separating abnormal and normal scans using PET as compared to SPECT. Increased effect size with PET as compared to SPECT has been reported previously by Mo and co-workers [25], and is likely caused by a more reliable delineation of putamen and caudate nucleus VOIs due to the increased spatial resolution of PET. Further, different reference regions for PET and SPECT may also account for some of the differences, and it must be stressed that variations between sites in delineations of striatum and reference regions may lead to differences in effect size. Lastly, we found similar effect size for semiquantitative measures using the shorter 30–40-min protocol as compared to the previously recommended 17–42-min protocol [25] supporting the use of a shorter scan time. The values using the 30–40-min protocol are a bit higher, which seems acceptable, as the 17–42-min protocol has been shown to slightly underestimate as compared to full dynamic imaging [25].

The strength of our study is the consecutive inclusion of unselected patients with a spectrum of abnormalities, including a high frequency of vascular changes and a good balance between normal and abnormal scans (42/56). Thus, the present findings are transferable to daily clinical routine.

Limitations include lack of follow-up data precluding final diagnoses of the patients, as a high fraction of the patients were referred for imaging from outpatient neurology clinics, precluding access to follow-up data. Instead, the study presents a direct comparison of [^18^F]FE-PE2I PET to the reference standard [^123^I]FP-CIT SPECT. A possible superiority of PET to SPECT was not feasible to assess with the present study design, although the increased effect size of putamen/caudate nucleus ratio is advantageous and could deliver a higher sensitivity using [^18^F]FE-PE2I PET as compared to [^123^I]FP-CIT SPECT in clinical use. The lack of a thorough description of the patients was due to highly variable clinical information provided by especially the outpatient neurological clinics, which could limit generalization. The limited or varying clinical data, however, reflects daily clinical routine and the authors do not believe that lack of clinical information will bias the results. Only a minority of the patients were suspected for atypical parkinsonism. Two additional limitations of the visual expert comparison include firstly the lack of supportive semiquantitative data for the PET evaluation with age-corrected thresholds based on healthy controls. Secondly, in a number of patients, no prior CT or MRI was available and the SPECT evaluation was performed without taking structural imaging into account in contrast to the PET reading which inherently included CT for all patients. The two latter limitations resulted in reduced agreement between modalities and our kappa value of 0.85 should be considered a conservative estimate. Indeed, half of the disagreement could be attributed to lesions identified using the CT obtained with PET in patients with no prior structural imaging. Thus, it must be stressed that the present comparison includes not only a comparison of tracers but also a comparison of availability of structural imaging, image analyses including region delineation, reference regions, etc., that may differ at different institutions.

In addition to a non-inferior diagnostic performance of [^18^F]FE-PE2I PET to the reference standard [^123^I]FP-CIT SPECT, several practical differences should be noted (Table 1). The use of [^18^F]FE-PE2I is preferable due to the higher selectivity for DAT. [^123^I]FP-CIT binding is susceptible to the use of selective serotonin reuptake inhibitors (SSRI) [11], although pre-scan drug withdrawal per se is not required in a clinical setting [38]. The improved logistics when using the shorter 30–40-min protocol with [^18^F]FE-PE2I PET as compared to [^123^I]FP-CIT SPECT decreases the overall time spent by the patients, their relatives and the staff (Table 1) and allows for an optimized and efficient use of scanner and waiting room facilities. Further, the use of PET technology instead of SPECT follows the trend of PET being an expanding field at the expense of SPECT. On the other hand, newer SPECT scanners may allow for shorter SPECT acquisitions reducing patient time in the scanner [41, 42], and the longer half-life of [^123^I]FP-CIT allows for on-site manufacturing/easier distribution for centres without access to a cyclotron. We have not compared costs due to varying pricing for PET tracer production that may be advantageous for either of the tracers depending on local factors.

Other PET tracers used for suspected neurodegenerative parkinsonian syndromes include the presynaptic tracers as [^11^C]PE2I, [^18^F]FDOPA, [^11^C]dihydrotetrabenazine and [^18^F]FP-DTBZ and postsynaptic tracers as [^18^F]fallypride, [^18^F]desmethoxyfallypride, and [^11^C]raclopride [38] of which [^18^F]FDOPA is the only tracer approved for clinical use by the US Food and drug administration and European Medicines Agency. No diagnostic accuracy studies have compared the efficiency of the PET tracers directly for diagnosing Parkinsonism, but a meta-analysis of 142 PET studies reports a consistently smaller PD-related decrease in tracers targeting the dopa decarboxylase such as [^18^F]FDOPA, as compared to tracers targeting the dopamine transporter or vesicular monoamine transporter [43] and thus suggests a smaller effect size for discrimination between PD and healthy controls using [^18^F]FDOPA compared to [^18^F]FE-PE2I PET. The difference is thought to reflect an upregulation of the dopa decarboxylase function in early PD and a downregulation of DAT, but [^18^F]FDOPA PET still shows excellent sensitivity and specificity for the diagnosis of PD [38].

## Conclusions

We find an excellent correspondence of [^18^F]FE-PE2I PET with the reference standard [^123^I]FP-CIT SPECT, and [^18^F]FE-PE2I PET is highly feasible for clinical use. Regarding patient comfort [^18^F]FE-PE2I PET/CT is outstanding compared to [^123^I]FP-CIT SPECT, especially when using the short-time protocol (30–40 min).

## Supplementary Information

**Additional file 1 **Table S1: STARD checklist. Contains the STARD checklist

**Additional file 2 **Table S2: Disagreement between [123I]FP-CIT SPECT and [18F]FE-PE2I PET. Table of patients classified different

**Additional file 3 **Figure S1: PET versus SPECT. Figure of SBR of each patient

## Data Availability

The datasets used and/or analysed during the current study are available from the corresponding author on reasonable request.

## Abbreviations

DAT: Dopamine transporter
PD: Parkinson’s disease
PS: Neurodegenerative parkinsonian syndromes
ROI: Regions of interest
HPLC: High-performance liquid chromatography
VOI: Volumes of interest
C.I.: Confidence interval
