# Correction: [^18^F]FE-PE2I PET is a feasible alternative to [^123^I]FP-CIT SPECT for dopamine transporter imaging in clinically uncertain parkinsonism

**DOI:** 10.1186/s13550-023-00970-x

**Published:** 2023-03-01

**Authors:** Lisbeth Marner, Kirsten Korsholm, Lasse Anderberg, Markus N. Lonsdale, Mads Radmer Jensen, Eva Brødsgaard, Charlotte L. Denholt, Nic Gillings, Ian Law, Lars Friberg

**Affiliations:** 1 Department of Clinical Physiology and Nuclear Medicine, Copenhagen University Hospital Bispebjerg, Bispebjerg Bakke 23, Copenhagen, Denmark; 2 Department of Clinical Medicine, University of Copenhagen, Copenhagen, Denmark; 3 Department of Clinical Physiology, Nuclear Medicine and PET, Copenhagen University Hospital Rigshospitalet, Copenhagen, Denmark

**Correction to: EJNMMI Research (2022) 12:56**

https://doi.org/10.1186/s13550-022-00930-x

Following publication of the original article [1], the following two errors in Table 2 came to the attention of the authors: the value '47' had been omitted from the row 'PS' ('Parkinsonian syndromes') and the title of the table contained the incorrect formatting '[18F]FE-PE2I' (that is, the number 18 had not been superscripted). The table has now been corrected in the published article, and the corrected table may be seen in this erratum.

**Table 2:**
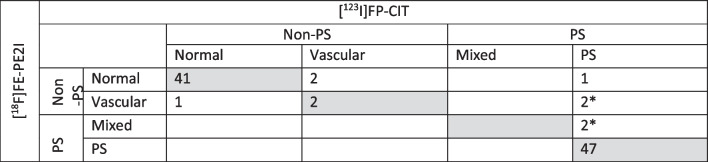
Agreement between [^123^I]FP-CIT and [^18^F]FE-PE2I

