# Supplementary material for: [18F]FE-PE2I PET is a feasible alternative to [123I]FP-CIT SPECT for dopamine transporter imaging in clinically uncertain parkinsonism
Source: EJNMMI Res. 2022 Sep 7;12:56. doi: 10.1186/s13550-022-00930-x (PMC9452620; doi:10.1186/s13550-022-00930-x)
Supplement: Supplementary file 2 — Additional file 2 Table S2: Disagreement between [123I]FP-CIT SPECT and [18F]FE-PE2I PET. Table of patients classified different [file 13550_2022_930_MOESM2_ESM.docx]

Supplementary Table 2. Disagreement between [^123^I]FP-CIT SPECT and [^18^F]FE-PE2I PET

| ***Pt no*** | ***[^123^I]FP-CIT SPECT*** | ***[^18^F]PE2I PET*** | ***Comment*** | ***Disagreement due to lack of prior imaging*** |
| --- | --- | --- | --- | --- |
| 1 | 2 | 0 | Prior CT showed infarction in basal ganglia bilaterally. The impact on PE2I-uptake was regarded less than impact on FP-CIT uptake.  The patient was diagnosed with vascular parkinsonism and later developed mild vascular dementia. |  |
| 2 | 0 | 2 | Prior MRI showed infarction in left basal ganglia. The impact on FP-CIT uptake was regarded less than impact on PE2I uptake.  The patient was later diagnosed with normal pressure hydrocephalus and a shunt relieved some of the symptoms. |  |
| 3 | 1 | 2 | No prior structural imaging, thus reduced FP-CIT was interpreted as PS. CT showed bilateral infarctions and the reduced PE2I uptake was interpreted as structural changes. Later MRI showed bilaterally bleedings in the basal ganglia. | yes |
| 4 | 1 | 2 | No prior structural imaging, thus reduced FP-CIT was interpreted as PS. CT showed infarction lateral for left putamen and the reduced PE2I uptake was interpreted as structural changes. No follow-up was available. | yes |
| 5 | 1 | 3 | No prior structural imaging, thus reduced FP-CIT was solely interpreted as PS. CT showed infarction in left caudate and putamen and the reduced PE2I-uptake was interpreted as mixed PS and structural changes | yes |
| 6 | 1 | 0 | Prior CT available. With better resolution, PE2I uptake was interpreted as normal as opposed to FP-CIT uptake which was interpreted as PS. Follow-up three years later showed continuous gait disturbances, increasing cognitive symptoms and normal PE2I uptake. Effect of Levodopa was described to be uncertain. |  |
| 7 | 2 | 0 | Prior CT showed infarcts in basal ganglia. The impact on PE2I-uptake was regarded less than impact on FP-CIT uptake. The patient was deteriorating and died within a year. |  |
| 8 | 1 | 3 | No prior structural imaging, thus reduced FP-CIT was solely interpreted as PS. CT showed bilateral infarctions and the reduced PE2I-uptake was interpreted as mixed PS and structural changes | yes |

0=normal, 1=PS, 2=structural, 3=mixed. PS: neurodegenerative parkinsonian syndrome.
