# Supplementary material for: [18F]FE-PE2I PET is a feasible alternative to [123I]FP-CIT SPECT for dopamine transporter imaging in clinically uncertain parkinsonism
Source: EJNMMI Res. 2022 Sep 7;12:56. doi: 10.1186/s13550-022-00930-x (PMC9452620; doi:10.1186/s13550-022-00930-x)
Supplement: Supplementary file 3 — Additional file 3 Figure S1: PET versus SPECT. Figure of SBR of each patient [file 13550_2022_930_MOESM3_ESM.docx]

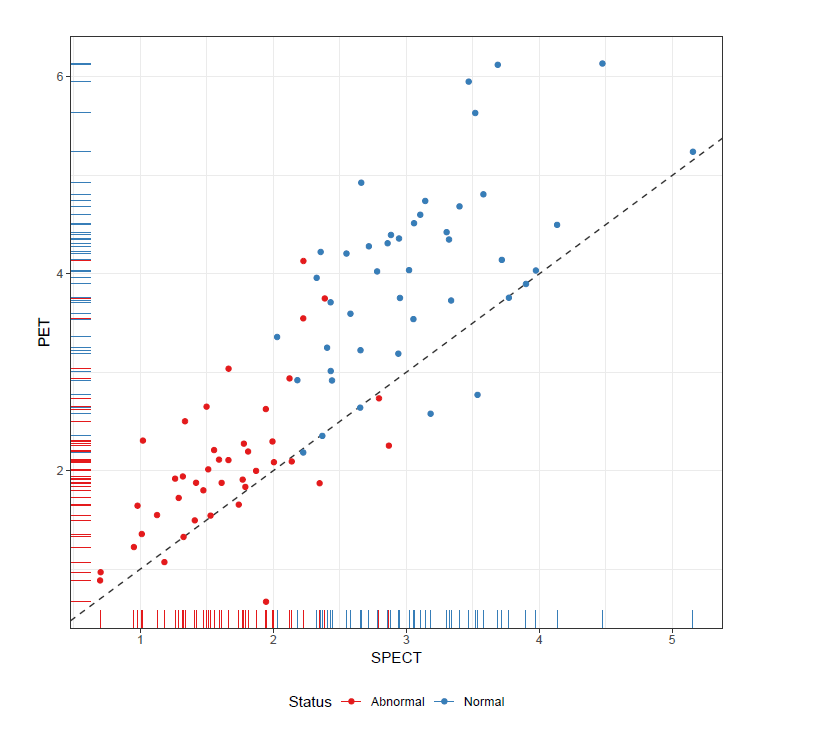


Supplementary Figure 1. PET versus SPECT.

The lowest putamen SBR of each subject, PET plotted against SPECT. The dotted line is the line of identity. Red circles are patients categorized as abnormal while blue are normal.
